# Cross-validated stepwise regression for identification of novel non-nucleoside reverse transcriptase inhibitor resistance associated mutations

**DOI:** 10.1186/1471-2105-12-386

**Published:** 2011-10-03

**Authors:** Koen Van der Borght, Elke Van Craenenbroeck, Pierre Lecocq, Margriet Van Houtte, Barbara Van Kerckhove, Lee Bacheler, Geert Verbeke, Herman van Vlijmen

**Affiliations:** 1 Tibotec-Virco, Beerse, Belgium; 2 I-Biostat, Katholieke Universiteit Leuven and Universiteit Hasselt, Belgium; 3 VircoLab Inc., Chapel Hill, NC, USA

## Abstract

**Background:**

Linear regression models are used to quantitatively predict drug resistance, the phenotype, from the HIV-1 viral genotype. As new antiretroviral drugs become available, new resistance pathways emerge and the number of resistance associated mutations continues to increase. To accurately identify which drug options are left, the main goal of the modeling has been to maximize predictivity and not interpretability. However, we originally selected linear regression as the preferred method for its transparency as opposed to other techniques such as neural networks. Here, we apply a method to lower the complexity of these phenotype prediction models using a 3-fold cross-validated selection of mutations.

**Results:**

Compared to standard stepwise regression we were able to reduce the number of mutations in the reverse transcriptase (RT) inhibitor models as well as the number of interaction terms accounting for synergistic and antagonistic effects. This reduction in complexity was most significant for the non-nucleoside reverse transcriptase inhibitor (NNRTI) models, while maintaining prediction accuracy and retaining virtually all known resistance associated mutations as first order terms in the models. Furthermore, for etravirine (ETR) a better performance was seen on two years of unseen data. By analyzing the phenotype prediction models we identified a list of forty novel NNRTI mutations, putatively associated with resistance. The resistance association of novel variants at known NNRTI resistance positions: 100, 101, 181, 190, 221 and of mutations at positions not previously linked with NNRTI resistance: 102, 139, 219, 241, 376 and 382 was confirmed by phenotyping site-directed mutants.

**Conclusions:**

We successfully identified and validated novel NNRTI resistance associated mutations by developing parsimonious resistance prediction models in which repeated cross-validation within the stepwise regression was applied. Our model selection technique is computationally feasible for large data sets and provides an approach to the continued identification of resistance-causing mutations.

## Background

Linear regression models have been shown to be accurate in predicting drug susceptibility from the HIV-1 viral genotype, by calculating the inhibitory concentration 50% (IC_50_) log Fold-Change (FC) phenotype as a linear combination of parameters, which are mutations [1-3] and interaction terms (mutation pairs) [1]. The coefficients of these parameters are named resistance weight factors (RWF), and they quantify the effect on the log FC of the mutations and mutation pairs. To generate models that are able to make predictions for future genotypes, ideally only resistance associated mutations are selected for the models. As it is not feasible to explore all possible subsets of mutations, stepwise regression is used to incrementally generate a series of regression models by addition or removal of mutations in each step. Different performance criteria exist to select one final linear regression model from this series [4,5]. In [1] standard stepwise regression was applied, selecting mutations based on significance with the F-test using predefined p-values. However, since correction for multiple significance-testing is not taken into account and because p-value thresholds are arbitrary, other selection criteria are preferred. Information criteria exist that balance accuracy and parsimony by penalizing for the number of parameters in the models. Information criteria commonly used are Akaike Information Criterion (AIC [6]) and Schwarz Bayesian Criterion (SBC [7]) with the penalty in SBC being more severe than in AIC. Although standard stepwise regression is a fast method in generating a model, the model found may be too complex by containing redundant information. Therefore, techniques are required that increase the stability of subset selection in linear regression. In [8] bootstrap aggregation ('bagging') was presented where an averaged prediction is made using multiple models generated on random re-samples of the original data set with replacement ('bootstrap') [9]. In [10] the bootstrap was integrated in the automatic selection procedure itself as parameters were sequentially added according to the proportion of bootstrap models in which they were selected. We investigated whether cross-validation [4,5] could be used as a less computationally intensive re-sampling technique than bootstrapping to reduce the complexity of the linear regression models while maintaining accuracy and adding to interpretability by generating only one regression model. As such, we aimed for an improvement in reliability of information extracted from the models, in this case the identification of novel mutations that cause resistance to anti-HIV drugs. In this article we use *Virtual*Phenotype™-LM (Virco, Beerse, Belgium) [1] as a reference prediction model. Since June 2006, *Virtual*Phenotype™-LM has been a linear regression model that predicts the log FC based on mutations (first-order terms) and mutation pairs (second-order interaction terms accounting for synergistic and antagonistic effects). We propose a more robust selection procedure by making two major changes to this reference approach. First, models were developed directly in second-order: interaction terms could be selected as soon as the constituting mutations were both present as first-order effect. Second, mutations or interaction terms were selected by repeatedly applying 3-fold cross-validation. In this article we refer to this method as 3F. Repeated cross-validation was presented as a way to reduce variability in the prediction error estimate [11]. Moreover, as shown in [12,13], repeated multi-fold cross-validation leads to better model selection when increasing the size of the validation set. To evaluate the generalizability of the models, the prediction error was calculated on genotypes in an unseen data set with available measured phenotypes.

After generating linear models with reduced complexity we were able to effectively identify novel mutations associated with NNRTI resistance. The individual contribution to resistance of these mutations was experimentally validated by making site-directed mutants and determining in vitro resistance levels.

## Results

### Reverse Transcriptase Inhibitors

**Figure 1:**
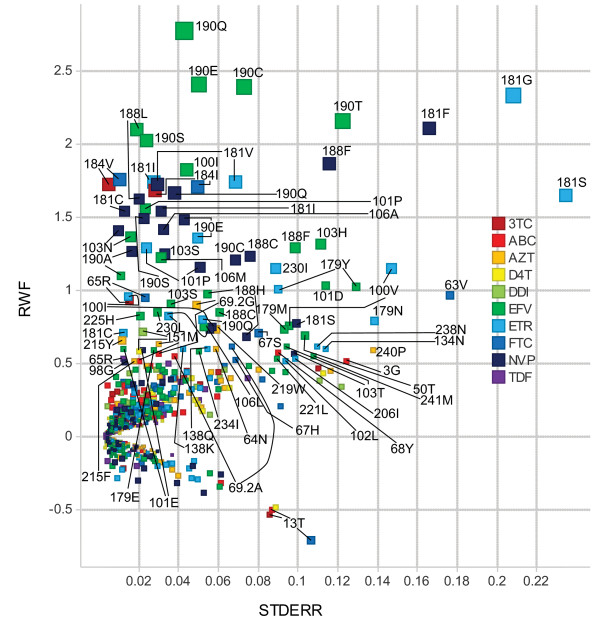
**RT mutations as first order effect in the 3F linear regression models**. RT mutations with their regression coefficient (RWF in log Fold Change) and standard error (STDERR) in the 3F linear models. Mutations with |RWF| ≥ 0.5 are labeled. RWF stands for Resistance Weight Factor (terminology adopted from *Virtual***Phenotype**™-LM).

For the reverse transcriptase inhibitors (RTI) a 3F model with lower complexity (using less mutations and less interaction terms) than the reference was found for AZT, 3TC, d4T, ABC, FTC, NVP, EFV and ETR (Table 1). For the nucleoside reverse transcriptase inhibitors (NRTI) class of drugs the reduction in interaction terms and mutations used in 3F versus reference was 20.3% and 11.9%, respectively. For the NNRTI class of drugs the 3F method was even more effective in reducing the complexity: the reduction in interaction terms and mutations was 38.3% and 26%, respectively. For all RTI with exception of AZT the 3F performance on unseen data equalled the reference (3F average squared error within 1% of the reference) or was better than the reference (for ETR: 3F average squared error 1.9% lower than in the reference) (Table 1). Moreover, the 3F model performance as compared to reference was maintained in subsets of unseen data samples, including the subset with one or more mutations included only in the reference model. Although the AZT 3F model had a lower SBC value on the training set than the reference, the averaged squared error on the unseen data was 1.5% higher in 3F. The reduction in the AZT 3F model compared to the reference of 28.7% interaction terms and 17.1% mutations thus resulted in 3F underfitting. Nevertheless, for AZT the concordance in susceptibility calls on the unseen data between 3F and reference was 94.67% and there were no 'major' discordances (fully susceptible or maximal response by reference but fully resistant or minimal response by 3F or vice versa) between the two approaches. In contrast, the ETR reference model had a lower SBC value on the training set than the 3F model but used 2.2 times more interaction terms and 1.3 times more mutations than the 3F model, thus implying reference overfitting. Here, the concordance in susceptibility calls between the two approaches was 90.56%, with one major discordance (see additional file 1: Comparison of susceptibility call between 3F and Reference on unseen data for ATV, AZT and ETR). An increase in the ratio of interaction terms versus single terms in a 3F RT inhibitor model had no significant impact on the 3F performance compared with the reference (P = 0.3673). In total, 172 and 196 different mutations were used as single terms in the NRTI and NNRTI 3F models, respectively (Figure 1, and see additional file 2: Complexity and performance of 3F and Reference models on genotype-phenotype data sequenced at Virco up to September 2006). The latter include all of the 44 NNRTI resistance-associated mutations identified in [14], with the exception of 179G, 227C, and 236L. Seventy-two RT mutations were present as single terms in both NRTI and NNRTI 3F models, including the highly prevalent mutations 103N and 184V. Three interaction terms were common between the AZT 3F model and one or more of the NNRTI 3F models: 103N&181C, 103N&184V and 215Y&219E. Some rare variants at RT position 181 were identified with high RWF and relatively high standard error: 181F, 181G and 181S (Figure 1).

**Table 1:** Complexity and performance of 3F and Reference models on genotype-phenotype data sequenced at Virco up to September 2006

		Reference Sep 2006			**3F Sep 2006**^**a**^			Unseen data		
								Sep 2006 - Dec 2008		
**drug**	***N***	**single**^**b**^	**int**^**c**^	**mut**^**d**^	**single**	**int**	**mut**	***N***	**ase**^**e**^	**ase**
	***train***	**terms**	**terms**		**terms**	**terms**		***test***	**Reference**	**3F**

Nucleoside RT inhibitors										
AZT	45734	80	108	123	66	77	102	8698	0.091	0.093
3TC	47422	59	64	70	43	52	45	8733	0.059	0.059
ddI	47269	49	21	62	50	25	54	8746	0.054	0.054
d4T	47235	47	34	68	54	20	60	8749	0.050	0.050
ABC	45908	71	46	90	63	24	68	8749	0.048	0.048
FTC	16440	31	35	46	34	34	36	8722	0.086	0.086
TDF	31640	64	91	110	79	83	111	8757	0.065	0.064
Nonnucleoside RT inhibitors										
NVP	47400	124	190	142	103	148	110	8729	0.101	0.100
EFV	46054	191	167	211	126	101	142	8687	0.266	0.264
ETR	18166	122	158	160	94	72	119	8493	0.126	0.124

^a^July-September genotype-phenotype 2006 data was used as validation set for 3F.

^b^Number of single terms (first order effects) in model.

^c^Number of interaction terms in model.

^d^Number of mutations in model.

^e^Average squared error on unseen genotype-phenotype data collected between September 2006 and December 2008.

**Figure 2:**
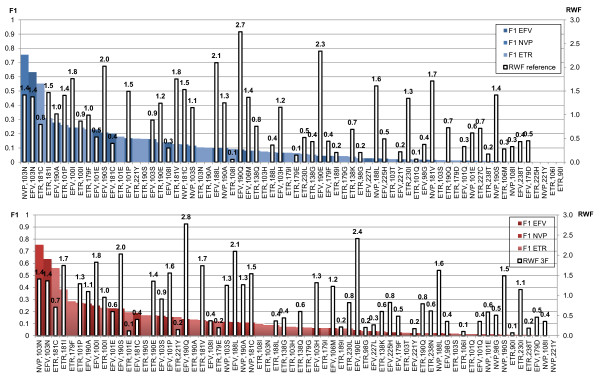
**Impact of known NNRTI resistance associated mutations**. LDA on reference (blue) and 3F (red) predicted phenotypes. Mutations shown are from the list of 44 known non-nucleoside RT inhibitor resistance associated mutations [14] having F1 > 0, ranked by F1.

Linear discriminant analysis (LDA [5,15]) was conducted to compare the predicted FC distribution of genotypes with a mutation from the list compiled in [14] to the genotypes having the wild-type amino acid at the corresponding position. Mutations with the highest impact (LDA F1 [16]) on NNRTI resistance are shown in Figure 2. Ranking by impact (LDA F1) was largely similar when comparing the 3F models to the reference, and the RWF were also very similar in both approaches. However, some clear differences between 3F and reference were observed. For example, for EFV, 190Q (the mutation with the highest RWF in reference and 3F) had more impact in 3F (F1 = 0.133 *vs*. 0.089). For ETR, mutation 179F had more impact in 3F than in the reference, although the mutation was not present as first-order effect in the 3F model (Figure 2). To detect novel NNRTI resistance-associated mutations a similar LDA was conducted for the remaining 100 RT mutations from the list of 124 found as first-order effect in the NNRTI 3F models but not in the NRTI 3F models. We ranked these novel mutations by their impact on NNRTI resistance, and retained the top 40 for further analysis as described in the next section.

### Novel resistance-associated mutations

Forty novel NNRTI resistance-associated mutations were found satisfying the following criteria: LDA cutoff > 0 (mutation contributes to resistance) for all of the NNRTIs, RWF > 0 in the 3F model of at least one of the NNRTIs, and RWF ≮ 0 in any 3F model of NVP, EFV or ETR (Table 2). The mutation with the highest impact (LDA F1) was 179M and occurred relatively rarely (31 times) in the LDA data set containing approximately 79,000 genotypes. Novel mutations that were more frequent with an impact > 0.01 were 376S, 138A and 357T. Mutation 138A has recently been recognized as an ETR resistance associated mutation [17]. Site-directed mutants were made for a selection of the novel mutations. Their resistance contribution was analyzed by looking at the mutant's FC relative to a drug-specific biological cutoff (BCO), used to separate viruses that are susceptible from those that show signs of resistance [18]. When comparing ETR to the other two NNRTIs in Table 2 in most cases the LDA F1 value for ETR was higher than for EFV and NVP (Table 2: max(F1)). However, the in vitro effect of the site-directed single mutations was, with exception of 138A, always below the BCO for ETR, while this was not the case for EFV and NVP. SDMs 100V, 190T, 101A and 101D had measured FC values above the BCO for NVP (> 6.0) and EFV (> 3.3). SDM 139R had an FC value above the BCO for NVP only. For the SDM 138A, the highest FC values measured were 4.9, (NVP, below BCO), 3.6, (EFV, above BCO) and 3.5, (ETR, above BCO (> 3.2)). Other mutations found with elevated FC for at least one of the NNRTIs were 221L, 219H, 219D, 376S, 102L, 101N, 234I, 382T, 139K and 241M.

**Table 2:** Novel non-nucleoside RT inhibitor resistance associated mutations

			**Site-directed Mutants**^**a**^			**LDA 3F**^**b**^			
				
**Mutation**^**c**^	***N wt***^**d**^	***N mut***^**e**^	NVP	EFV	ETR	max(*F*1)	**max(*n***_**11**_**)**^**f**^	$\max{\left(\frac{n_{11}}{n_{11}+n_{01}}\right)}^{\text{g}}$	**min(*cutoff*)**^**h**^
**V179**M	67699	31	*0.8-2.3*	*0.3-0.4*	*0.4-0.9*	0.327 (EFV)	13 (ETR)	8/(8 + 10) (EFV)	1.35 (ETR)
**H221**L	76783	31	*4.2-5.0*	*1.5-1.6*	*1.1*	0.118 (EFV)	2 (EFV)	2/(2 + 1) (EFV)	2.03 (ETR)
**V179**Y	67699	17	*0.2-0.5*	*< 0.2-0.2*	*< 0.1-0.2*	0.063 (ETR)	16 (ETR)	16/(16 + 472) (ETR)	1.44 (ETR)
K219H	67913	129	*1.3-2.2*	*1.0-1.8*	*1.1-1.8*	0.047 (ETR)	14 (ETR)	14/(14 + 447) (ETR)	1.45 (ETR)
K219D	67913	161	*1.4-3.8*	*1.3-2.2*	*0.4-2.0*	0.044 (ETR)	24 (ETR)	24/(24 + 894) (ETR)	1.22 (ETR)
**V179**N	67699	15	*1.2-1.9*	*0.9-1.2*	*0.6-0.8*	0.039 (ETR)	8 (ETR)	8/(8 + 391) (ETR)	1.50 (ETR)
T376S	15617	7415	*2.8-3.4*	*1.5-2.0*	*1.6-2.1*	0.035 (EFV)	135 (EFV)	135/(135 + 145) (EFV)	1.67 (ETR)
**Y181S**	72643	3	NA	NA	NA	0.019 (ETR)	3 (ETR)	3/(3 + 310) (ETR)	1.45 (ETR)
**L100**V	77197	8	**6.4 (1)**	**9.0-18.3 (2)**	*1.1-1.2 (2)*	0.017 (ETR)	6 (ETR)	6/(6 + 678) (ETR)	1.63 (ETR)
**Y181**F	72643	9	*1.1-2.0*	*0.4-0.8*	*0.4-0.7*	0.016 (ETR)	4 (ETR)	4/(4 + 503) (ETR)	1.33 (ETR)
K102L	72791	12	*2.3-2.9*	*0.5-1.2*	*0.3-0.4*	0.015 (ETR)	4 (ETR)	4/(4 + 505) (ETR)	1.77 (ETR)
**K101**N	72175	94	*5.0 *(1)	*2.8 (1)*	*0.8 (1)*	0.015 (ETR)	4 (ETR)	4/(4 + 434) (ETR)	1.66 (ETR)
**V106**L	75529	44	*0.5-0.6*	*0.7-1.1*	*0.2-0.3*	0.015 (ETR)	5 (ETR)	5/(5 + 617) (ETR)	1.69 (ETR)
**E138**A	75869	1828	*2.2-4.9*	*1.2-***3.6**	*2.8-***3.5**	0.014 (ETR)	14 (ETR)	14/(14 + 157) (ETR)	2.10 (ETR)
M357T	44115	17866	*0.9 (1)*	*1.2 (1)*	NA	0.013 (ETR)	117 (ETR)	117/(117 + 199) (ETR)	1.88 (ETR)
T139R	76899	243	**6.4-7.3**	*1.5-2.7*	*1.0-1.2*	0.010 (ETR)	2 (ETR)	2/(2 + 137) (ETR)	2.22 (ETR)
E370G	75915	489	NA	NA	NA	0.007 (ETR)	2 (ETR)	1/(1 + 1) (EFV)	2.38 (ETR)
**I135T**	45829	18410	NA	NA	NA	0.006 (ETR)	52 (ETR)	52/(52 + 94) (ETR)	2.16 (ETR)
**L234I**	79037	98	*0.6-1.0*	*1.6-2.3*	*0.9-1.1*	0.004 (ETR)	1 (ETR)	1/(1 + 353) (ETR)	1.95 (ETR)
S379C	69973	3578	NA	NA	NA	0.001 (ETR)	1 (ETR)	1/(1 + 1) (ETR)	3.35 (ETR)
R206I	79051	8	*1.0-1.7*	*0.4-0.7*	*0.5-0.9*	NA	0 (ETR)	0/(0 + 102) (ETR)	2.33 (ETR)
S134N	79041	19	*1.2-2.1*	*0.6-0.7*	*0.8-0.9*	NA	0 (ETR)	0/(0 + 69) (ETR)	2.45 (ETR)
**H221**C	76783	59	NA	NA	NA	NA	0 (ETR)	0/(0 + 20) (ETR)	2.73 (ETR)
I382T	78025	329	*2.4-***6.7**	*0.9-1.7*	*0.7-2.4*	NA	0 (ETR)	0/(0 + 2) (ETR)	3.30 (ETR)
D237E	78246	423	NA	NA	NA	NA	0 (EFV)	0/(0 + 3) (EFV)	3.67 (ETR)
N348T	74372	170	NA	NA	NA	NA	0 (ETR)	0/(0 + 0) (ETR)	4.05 (ETR)
E399G	66049	670	NA	NA	NA	NA	0 (ETR)	0/(0 + 0) (ETR)	4.10 (ETR)
**G190**T	72912	10	**> 67.4**	**7.8-14.9**	*0.6-0.7*	NA	0 (EFV)	0/(0 + 2) (EFV)	4.16 (ETR)
**Y188**F	76892	41	*1.4-1.9*	*0.3-0.5*	*0.2-0.6*	NA	0 (NVP)	0/(0 + 0) (NVP)	4.70 (NVP)
**L283I**	72462	5930	NA	NA	NA	NA	0 (ETR)	0/(0 + 0) (ETR)	5.01 (ETR)
**K101**A	72175	50	**8.8-13.4**	**4.1-5.6**	*1.5-1.8*	NA	0 (EFV)	0/(0 + 3) (EFV)	5.04 (NVP)
**K101**D	72175	7	**13.3-18.9**	**5.7-6.8**	*1.0-1.3*	NA	0 (EFV)	0/(0 + 3) (EFV)	5.08 (EFV)
T139K	76899	348	*4.4-5.8*	*1.2-2.3*	*2.4-3.0*	NA	0 (ETR)	0/(0 + 0) (ETR)	5.10 (ETR)
T165L	75078	183	NA	NA	NA	NA	0 (ETR)	0/(0 + 0) (ETR)	5.81 (ETR)
**T386A**	59810	1756	NA	*1.6 (1)*	*0.5 (1)*	NA	0 (NVP)	0/(0 + 0) (NVP)	6.07 (NVP)
V241M	78771	23	*4.7-5.6*	*1.0-1.8*	*0.8-1.2*	NA	0 (NVP)	0/(0 + 0) (NVP)	6.90 (NVP)
I382L	78025	228	NA	NA	NA	NA	0 (NVP)	0/(0 + 0) (NVP)	7.30 (NVP)
G335S	65035	1877	NA	NA	NA	NA	0 (ETR)	0/(0 + 0) (ETR)	7.55 (ETR)
**E399D**	66049	10697	NA	NA	NA	NA	0 (ETR)	0/(0 + 0) (ETR)	7.98 (ETR)
R358K	70517	5995	NA	NA	NA	NA	0 (NVP)	0/(0 + 0) (NVP)	8.06 (NVP)

^a^Fold-Change range from 3 measurements, unless otherwise indicated between brackets.

FC > Biological Cut-Off (BCO) in bold, FC ≤ BCO in italic; BCO for NVP is 6.0, BCO for EFV is 3.3 and BCO for ETR is 3.2.

^b^Summarized for the three non-nucleoside RT inhibitors (NNRTI).

^c^Top 40 mutations, ranked by max(LDA F1) descending, then by min(LDA cutoff) ascending. Mutations shown are from the list of 124 NNRTI mutations with RWF ≥ 0 and LDA cutoff > 0 for NVP, EFV and ETR. Known NNRTI positions or novel mutations listed in [32,33] are shown in bold.

^d^Frequency of wild-type (not within a mixture) in LDA data set.

^e^Frequency of mutation (not within a mixture) in LDA data set.

^f^*n*_11 _is the number of samples with amino acid mutation having a predicted phenotype above the LDA cutoff.

^g^*n*_01 _is the number of samples with wild type amino acid having a predicted phenotype above the LDA cutoff.

^h^Cutoff in log Fold-Change (taking the wild-type and mutation frequency percentages as prior probabilities in the LDA can result in cutoff values outside of the range of the predicted phenotypes).

As these novel mutations frequently co-occur with known resistance-associated mutations, we also studied their effect in genetic background containing such mutations. The Virco database of clinical isolates was searched for the most appropriate genetic backgrounds to test each of the novel mutations for their effect on NNRTI resistance [19]. For the mutations 139R, 219D and 219H we looked at their contributions to resistance in combination with the highest-impact NNRTI resistance mutants 103N and 181C (Figure 2, and see additional file 3: Linear Discriminant Analysis (LDA) for 103N and 181C). The following site-directed mutants were tested for all NNRTIs: 103N, 103N+181C, 139R+103N+181C, 219D+103N+181C and 219H+103N+181C (Table 3). While for NVP and EFV all of the above combinations were resistant, for ETR the single mutation 103N and the combination 103N+181C were susceptible with mean FC values of 0.9 and 3.0, respectively. The limited impact (3F LDA F1: 0.09) of 103N on ETR resistance was thus confirmed by the site-directed mutant and adding 181C did not result in a FC above the BCO. Remarkably, all the above triple combinations were found to be resistant for ETR, thereby clearly demonstrating the contribution to NNRTI resistance of the novel mutations. For ETR a 2.2, 6.3 and 4.6-fold increase in FC compared to 103N+181C was seen when adding 139R, 219D and 219H, respectively. For EFV a 1.8-2.6-fold increase was seen as well. For NVP the contribution to resistance of the novel mutations in combination with 103N+181C could not be confirmed due to IC_50 _values larger than the maximum assay concentration (Table 3). Data for the novel mutations 102L, 138A, 139K, 139R, 179Y, 181F, 188F, 221L and 234I tested in different genetic backgrounds can be found in additional file 4: Site directed mutants of novel mutations tested for NVP, EFV and ETR, extending Table 2 with results of combinations.

**Table 3:** Site-Directed Mutants of novel NNRTI resistance associated mutations 139R, 219D and 219H in combination with 103N+181C and SDM 181G

SDM	drug	**3 measurements (Fold Change**^**a**^**)**		
139R	NVP	**7.3**	**7.3**	**6.4**
	EFV	*1.5*	*2.2*	*2.7*
	ETR	*1.2*	*1.2*	*1.0*

219D	NVP	*3.7*	*3.8*	*1.4*
	EFV	*1.3*	*2.2*	*1.3*
	ETR	*1.1*	*2.0*	*0.4*

219H	NVP	*2.1*	*2.2*	*1.3*
	EFV	*1.1*	*1.0*	*1.8*
	ETR	*1.8*	*1.7*	*1.1*

103N	NVP	**> 51.7**	**> 54.2**	**35.4**
	EFV	**19.5**	**15.1**	**12.5**
	ETR	*1.1*	*0.9*	*0.6*

103N+181C	NVP	**> 85.9**	**> 85.9**	**> 85.9**
	EFV	**28.4**	**23.1**	**38.7**
	ETR	**4.2**	*1.7*	*3.2*

139R+103N+181C	NVP	**> 22.7**	**> 21.8**	**> 22.7**
	EFV	**60.2**	**77.0**	**61.2**
	ETR	**7.0**	**7.4**	**5.6**

219D+103N+181C	NVP	**> 79.6**	**> 79.6**	**> 76.7**
	EFV	**116.6**	**72.9**	**48.6**
	ETR	**15.5**	**20.2**	**21.4**

219H+103N+181C	NVP	**> 79.6**	**> 79.6**	**> 79.6**
	EFV	**92.6**	**23.7**	**49.0**
	ETR	**14.5**	**16.1**	**11.6**

181G	NVP	**30.1**	**38.3**	**> 37.2**
	EFV	*1.6*	*1.3*	*1.1*
	ETR	*1.5*	*0.9*	*1.2*

^a ^> Biological Cut-Off (BCO) in bold, ≤ BCO in italic; BCO for NVP is 6.0, BCO for EFV is 3.3 and BCO for ETR is 3.2.

Mutation 181G was not in the list of novel mutations as it was always found in presence of other amino acids at position 181 (a 'mixture') in the LDA data set. A site-directed mutant, containing 181G only, was made and found to be resistant to NVP (FC: 30.1-38.3) and having FC values of 1.1-1.6 for EFV and 0.9-1.5 for ETR (Table 3).

## Discussion

In order to quantitatively predict drug resistance from the HIV-1 viral genotype we used 3-fold cross-validation for the stepwise selection of model mutations or mutation pairs. Importantly, we applied a random division in three parts before each removal step (see additional file 5: K Fold cross-validated stepwise regression using same or different random division before each removal step: ETR model). Thus, in the 3F method robustness was achieved by removing model parameters that were not consistently selected under different random divisions. As a consequence, the 3F method can be considered a greedy stepwise approach where the probability of a parameter being removed increases as the model size increases. As we had to generate models for several drugs this computational approach was a practical one for our purpose. Nevertheless, to make the model selection less greedy, one might consider repeating the cross-validation at each step multiple times with a different random division in three parts, to improve the estimation of the prediction error.

As our goal was to reduce the complexity and maintain the accuracy of the reference models, we did not investigate defining a stop criterion for the 3F method independent from the reference. Instead, we retained all 3F models with better performance (AIC/SBC) than the reference on the genotype-phenotype data, holding out the two last months. The 3F model with best performance on the hold-out set was then selected as the final model, and the 3F model parameters were recalculated on the genotype-phenotype data including the hold-out set. Ultimately, in deciding between different approaches, the evaluation of performance on future observations remains the first priority. Therefore we tested the performance of the reference and 3F method on a large unseen genotype-phenotype data set sequenced at Virco between September 2006 and December 2008. For the RT inhibitors the 3F method maintained the accuracy of the reference models while reducing complexity of the linear regression models.

We also evaluated the performance of the 3F method for protease inhibitors (see additional file 2: Complexity and performance of 3F and Reference models on genotype-phenotype data sequenced at Virco up to September 2006). An equal performance between 3F and reference was seen for the most recent protease inhibitors TPV and DRV, and with both methods the models were less complex than for the other PIs. For the older protease inhibitors the performance of the reference approach was better than the 3F approach. This suggests that protease inhibitors for which resistance patterns have become more complex over time also require more complex regression models, thus increasing the importance of interaction terms. This is in conflict with the constraint in the 3F method that allowed only interaction terms of which both first order effects were already present in the model during the stepwise regression.

For the identification of resistance-associated mutations we restricted our attention to the NNRTIs. We detected novel variants at NNRTI resistance-associated positions 100, 101, 179, 181, 188, 190, 221, as well as at novel NNRTI resistance-associated positions 102, 139, 219, 241, 376 and 382.

As genotyping was done up to RT amino acid (AA) position 400, the 3F linear models of the RTIs, with exception of 3TC and FTC, also contained connection domain mutations (AA 289-423 of RT). Two out of eight of the connection domain mutations described in [20] to be associated with AZT resistance, were found to increase resistance as single term in the AZT 3F model: 348I and 360V. In [21], 376S was found to be associated with an increased risk of virological failure to NVP-based therapy in NNRTI-naïve patients and we could confirm the in vitro effect on NNRTI resistance by site-directed mutagenesis. In [22], resistance predictions for AZT, NVP and EFV by current genotypic drug resistance interpretation systems were found to be non-inferior when predicting from short RT sequences (AA 41-238 for RT) compared to full RT sequences. Nevertheless, our analysis suggests that there are 12 novel NNRTI resistance associated connection domain mutations, including 376S and 382T, that could provide more accurate predictions of NNRTI drug resistance.

Mutations found as a single term in both NRTI and NNRTI 3F models consisted of mutations at NRTI positions associated with NNRTI hypersusceptibility [23-25], including 215Y, found to be ETR hypersusceptible in the reference and 3F models, of connection domain mutations, including 348I, and of NNRTI resistance associated mutations from the list compiled in [14]. Our 3F models suggest that these latter mutations might have an effect on NRTI resistance as well (two have already been confirmed to have such an effect: 100I [26] and 181C [27]). In this study we were interested in novel NNRTI resistance associated mutations and we considered RT mutations found in the NNRTI 3F models only.

By making site-directed mutants we compared the resistance effect of mutations as first-order effect in the models with the in vitro effect. These results show that despite the reduction in model complexity for the 3F approach, one must still be cautious when equating linear model coefficients with in vitro effect. For example, mutation 181F has only a small in vitro effect for NVP whereas in the 3F regression model this mutation has a high first-order coefficient. A likely explanation for this is that in the same 3F model interaction terms of 181F with 98G, 103N, 106M, 190A (all known resistance key mutations [14]) with a strong predicted resensitizing effect are present as well, canceling out the resistance contribution of 181F as first-order effect. Specifically, we could confirm the resensitizing effect of the combination 181F+103N by comparing the resistance levels of SDM 181F+103N and SDM 103N. (see additional file 4: Site directed mutants of novel mutations tested for NVP, EFV and ETR). The systematic co-occurrence of 181F with known resistance mutations probably resulted in a high ranking for mutation 181F in the list of novel NNRTI resistance associated mutations.

As another example for ETR 179F is present as a first-order effect with a large resistance coefficient in the reference model whereas in the 3F model 179F is not present as a first-order effect. Inspecting this further we found the interaction term 179F&181C to be present with a strong resistance effect in the 3F model, but absent in the reference model. In this case the 3F method was in line with in vitro selection experiments done in [28], where the synergistic effect of 181C and 179F on the decreased susceptibility of ETR was already described. Moreover, the one genotype with measured resistant phenotype that resulted in a major discordance for ETR between 3F (Minimal Response) and reference (Maximal Response) contained exactly the 179F&181C combination (see additional file 1: Comparison of susceptibility call between 3F and Reference on unseen data for ATV, AZT and ETR). Most of the novel derived NNRTI resistance associated mutations had the highest impact (LDA F1 value) on resistance for ETR but a relatively low in vitro effect in comparison with NVP and EFV. Co-occurrence of multiple resistance associated mutations is needed to cause an elevated ETR FC, as exemplified by the SDMs containing two known resistance associated mutations and one novel mutation (e.g. 139R+103N+181C) with FC values above the BCO for ETR.

## Conclusions

By applying repeated 3-fold cross-validation within the stepwise regression, we could lower the complexity of linear regression models for predicting drug resistance while retaining performance on unseen data. The described 3F method thus proves to be a tractable approach when interpretation of the linear model is an objective. As the 3F method worked particularly well for the non-nucleoside reverse transcriptase inhibitors, we derived a list of forty novel NNRTI resistance associated mutations. For a selection of the novel mutations we confirmed their in vitro contribution to resistance by site-directed mutagenesis, individually or in combination with known resistance mutations. As most of these novel mutations were found at relative low frequency in patient samples, carefully following up on drug resistance in patients with viruses carrying these mutations may provide more insight in understanding how the virus escapes the current antiretroviral treatment, as well as in the design of novel drugs.

## Methods

### Genotype-phenotype database

The size of the data sets of genotypes (AA 1-99 of Protease (PR) and AA 1-400 of RT) with an available measured phenotype (Antivirogram, Virco) ranged from approximately 20,000 to 56,000 samples for all protease and reverse transcriptase inhibitors. Phenotypes were measured as the log FC in IC_50 _of a sample relative to a wild-type laboratory reference strain.

### Reference Linear Models

**Figure 3:**
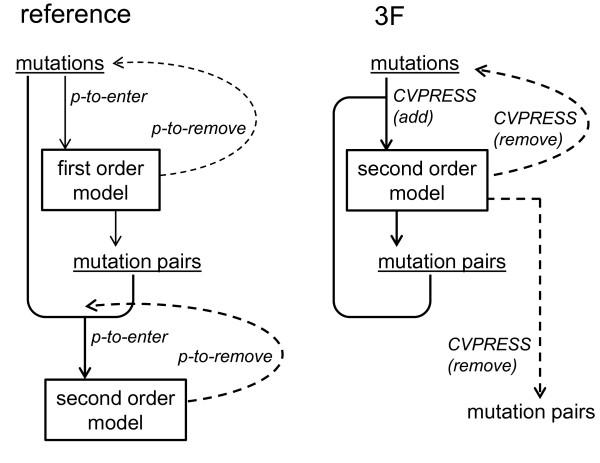
**Reference and 3F methodology: schematic overview**. In the 3F method cross-validated prediction error (CVPRESS) was used instead of significance levels (p-values) in the reference approach. In the reference approach two stepwise regression procedures were used: all possible mutation pairs were made from mutations in the first order model and candidate for entry in the second order model. In the 3F method, the initial search space consisted of all individual mutations. Mutation pairs could only enter the model if both mutations in the pair were already selected for the model.

*Virtual*Phenotype™Linear Models were calculated for all drugs on samples sequenced at Virco up to July 2006 and recalculated 2 months later (samples up to September 2006). Two stepwise regression procedures were used to develop the models [1]. First, a model was calculated using single mutations only. In the second stepwise regression procedure, all possible interaction terms containing the mutations present in the first-order model were also considered for selection, to account for synergistic and antagonistic effects. Cutoff values for p-values were used to determine which mutations or interaction terms could enter the model (SLE) or stay in the model (SLS) (Figure 3). The minimal number of occurrences in the database for mutations or interaction pairs to be considered in the model was 10 for FTC, ETR, ATV, TPV and DRV and 20 for all other drugs, limiting the number of mutations considered to approximately 300 for PR inhibitors and 1000 for RT inhibitors. Mutations occurring in mixtures (ambiguous sequencing results) were weighted accordingly, mixtures of more than four amino acids were not considered. No validation set was used in selection of the reference models.

### 3F Linear Models

In the 3F method three fold cross-validated prediction error sum of squares (CVPRESS) was used as selection criterion for parameter inclusion, instead of the significance levels (SLS and SLE) used in the reference approach. The motivation for this choice of fold is described in the next section. During 3-Fold cross-validation the data set is randomly split in three equal-sized parts, and a model is generated three times on two of the three parts and validated/tested on the remaining part. In this repetitive modeling each part is used only once for validation so that every genotype is treated once as unseen. A mutation was selected based on the performance (prediction error) calculated for the 'unseen' genotypes. We applied this cross-validation directly in each step of the selection procedure in evaluating which parameter to add or which parameter(s) to remove from the model. The initial search space consisted of all individual mutations. As soon as two mutations at different positions were selected, the cross-term of both could enter the model (we did not force this hierarchy constraint in the removal step, meaning that mutations could be removed as first-order effect while still present in an interaction term). The search space was thus dynamically enlarged with interaction terms as more single mutations were added (Figure 3). Models were calculated on the July 2006 data set. The same minimal counts (10/20) as for building the July 2006 reference model were used as thresholds for mutations to enter the 3F model. No minimal count was set for incorporation of interaction terms, as the 3F method already in concept selected for less interaction terms. The CVPRESS selection criterion can be specified directly in the SAS GLMSELECT procedure [29]. Therefore CVPRESS can be applied in the same way as selection criteria like AIC or SBC.

### Randomized stepwise selection

By using CVPRESS as selection criterion, a choice of fold K had to be made. We selected ETR as drug for evaluating different K. In short, the results of this evaluation were as follows (see additional file 5: K Fold cross-validated stepwise regression using same or different random division before each removal step: ETR model). Keeping the same random division throughout the stepwise regression, K = N (Leave-One-Out, also called PRESS [4]) was found too costly to compute [5,30]. Moreover, as the N training sets were almost similar, the improvement on the reference was rather a result of the hierarchy constraint for introduction of interaction terms than of the cross-validation itself. Nevertheless, choosing K < N automatically leads to selection bias. For K = 3, an apparent decrease in the ratio of (SBC_CV _- SBC) over the number of model parameters was observed as the model size increased, suggesting overfitting. Therefore, to generate better generalizing models, we decided to alter the random division before each removal step in the stepwise regression. Moreover, we now found K = 3 to be the best choice of fold in generating a model with lower SBC than the reference, and using the lowest number of parameters. Choosing K = 2, the required performance, set by the reference, could not be achieved using a limited number of random divisions, due to an increase of the number of parameter removals. Consequently, for K = 2, also the difference between SBC_CV _and SBC was found to be the largest, indicating that parameter selection became more difficult when only half of the samples was used for training of the model.

The randomized stepwise selection procedure can now be described. An initial forward stepwise regression was executed until two mutations at different positions were present in the model. After that the stepwise selection procedure amounted to the execution of multiple backward-forward regression steps cycles, with each cycle consisting of a backward/removal step followed by a forward/addition step. To avoid overfitting, a different random division in three parts was used in each of these cycles. As a consequence of this randomization, it was possible that the latest parameter added in the forward step of cycle *i *was directly removed in the backward step of cycle *i+1*, for instance when a model parameter was only present in one of the three parts of the new random division. This was in fact the reason why we decided to alter the random division before each backward step and not before each forward step. Cross-validation thus penalized infrequent interaction terms to get/stay in the model but was nevertheless less stringent than applying a minimal count rule as was done in the reference method.

### 3F model selection

The Virco genotype-phenotype data set between July and September 2006 was used as validation/test set. The 3F models were then selected as follows (Table 4): i) select all models with a better performance than the reference model on the training set with either AIC or SBC and ii) from the models selected in i), select the model that has the best performance (average squared error) on the test set. The selected 3F models were then refitted on all samples (including the validation set) to actually compare them to the September 2006 reference models.

**Table 4:** 3F model selection on genotype-phenotype data up to September 2006

		**3F Model generation**^**a**^			**3F Model selection**^**b**^				
			
drug		# 3F Models	# lower SBC	# lower AIC	**lower SBC**^**c**^	**lower AIC**^**d**^	*N test*	ase	**3F model selected**^**e**^
Nucleoside RT inhibitors^f^	AZT	300	86	0	yes	no	800	0.103	296
	3TC	150	60	34	yes	no	807	0.037	99
	ddI	150	20	70	no	yes	807	0.049	83
	d4T	120	41	35	yes	no	806	0.040	81
	ABC	200	111	53	yes	no	807	0.038	95
	FTC	80	28	22	yes	yes	804	0.071	76
	TDF	400	66	196	no	yes	807	0.039	298

NNRTI^g^	NVP	400	93	0	yes	no	801	0.089	391
	EFV	500	101	0	yes	no	807	0.246	386
	ETR	700	49	0	yes	no	777	0.113	656

Protease inhibitors	IDV	485	50	51	yes	yes	805	0.075	482
	NFV	375	64	6	yes	yes	808	0.063	375
	SQV	600	53	0	yes	no	807	0.092	575
	APV	1000	0	656	no	yes	808	0.060	709
	LPV	500	205	28	yes	no	807	0.157	319
	ATV	1275	0	2	no	yes	805	0.117	1158^h^
	TPV	1000	641	142	yes	no	806	0.059	428
	DRV	1000	823	799	yes	yes	816	0.096	707

^a^The number of 3F models generated was arbitrary but taken large enough such that at least one 3F model was found with a lower SBC or AIC than the reference on the genotype-phenotype data set up to July 2006.

^b^From the remaining 3F models with lower SBC or AIC than the reference, the 3F model was then selected with the lowest average squared error (ase) on an unseen genotype-phenotype data set collected between July and September 2006 (test set) containing approximately 800 samples.

^c^SBC of the selected 3F model < SBC reference on the test set (yes/no).

^d^AIC of the selected 3F model < AIC reference on the test set (yes/no).

^e^The number of different random divisions used in the stepwise regression in the selected 3F model.

^f^For the nucleoside RT inhibitors the number of random divisions needed was less than 100, with exception of AZT and TDF.

^g^For the non-nucleoside RT inhibitors most random divisions were needed for ETR.

^h^ATV was the only drug for which more than 1000 different random divisions were needed.

AIC is defined as *n *ln(*SSE*/*n*) + 2*p*. SBC is defined as *n *ln(*SSE*/*n*) + ln(*n*)*p*. In the above definitions *SSE *is the Sum of Squared Errors, *n *is the number of genotypes-phenotypes and *p *is the number of parameters in the linear regression model [4].

### Linear Discriminant Analysis

To rank RT mutations by their impact on NNRTI resistance, a linear discriminant analysis (LDA) was done on approximately 79,000 predicted phenotypes calculated from genotypes sequenced at Virco between September 2006 and December 2008. Note that the LDA was done on the calculated phenotypes and not on the measured phenotypes in order to incorporate as many recent genotypes as possible, especially since some of the novel NNRTI mutations are infrequent in occurrence (Table 2). In order to analyze the impact of a mutation at position *i *on resistance, a division of genotypes into two groups was made. The first group contained all genotypes containing the wild-type (HXB2) amino acid at position *i *and the second group the genotypes with the amino acid mutation. Genotypes containing a mixture of amino acids at the mutated position were not considered. A contingency table was then made to analyze how well the two groups could be separated using the LDA cutoff applied on the calculated phenotypes. The following metrics were calculated on the two-by-two contingency table: precision, recall and F1. Precision is defined as $\frac{n_{11}}{n_{11}+n_{01}}$. Recall is defined as *n*_11_/*N*_1_. *n*_11 _is the number of samples with the amino acid mutation having a calculated phenotype above the LDA cutoff. *n*_01 _is the number of samples with the wild-type amino acid having a calculated phenotype above the LDA cutoff. *N*_1 _is the number of samples with the amino acid mutation. As precision (positive predictive value) of the LDA discrimination in log FC of the mutated group from the wild type group at a position *i *can be high, while recall (sensitivity) is low or vice versa, we used the F1 metric, trading off precision and recall, for the ranking of mutations for their impact on resistance. F1 is defined as $\frac{\left(2\times p\times r\right)}{\left(p+r\right)}$ and thus equally weights precision (*p*) and recall (*r*). Ranking by impact on resistance (F1) was done for the known NNRTI resistance-associated mutations. For novel mutations, exclusively present as first-order effect in the 3F NNRTI linear regression models (thus absent in 3F nucleoside reverse transcriptase linear regression models), ranking for being associated with resistance was done using F1 if *p *+ *r *> 0 and by LDA cutoff otherwise. LDA analysis was done for both the reference and 3F calculated phenotypes calculated using the September 2006 models.

### Site-Directed Mutants

Site-directed mutants were created at Eurofins Medigenomix GmbH (Ebersberg, Germany) using the linear reaction method. In this method, the template DNA is linearly amplified using a mutagenesis-grade high-fidelity DNA polymerase which extends the mutagenic primers containing the desired mutation, incorporating the mutation of interest into the newly synthesized strands. The unique primer design allows replication of only the parental strand. Final treatment with Dpn I ensures the digestion of only dam-methylated parental strands. The resulting mutagenic strands were then transformed in ultracompetent cells and cultured on an agar plate. Single colonies were sequenced to ensure the availability of the correct mutation in the strand. A colony of a correct mutation containing strand was cultured and the purified plasmid shipped to Virco. Starting from this plasmid, the Protease - Reverse transcriptase region (AA 1-99 of PR and AA 1-400 of RT) was amplified and transfected into 293T cells and recombined with the deletion backbone by homologous recombination [31]. The cultivated virus was then grown against a standard set of anti-HIV drugs.

## Authors' contributions

KVdB designed the linear regression modeling approach, performed statistical analyses to derive a list of novel resistance-associated mutations, and drafted the manuscript. EVC and PL conceived of the study and assisted in its design. MVH assisted in interpreting the results and critically revised the manuscript. BVK coordinated the Virco lab work of the site-directed mutants and interacted with Medigenomix. LB participated in study design. GV advised on statistical analyses and helped draft the manuscript. HvV contributed to the design of the study and edited the manuscript. All authors read and approved the final manuscript.

## Supplementary Material

Additional file 1 **Comparison of susceptibility call between 3F and Reference on unseen data for ATV, AZT and ETR**. For ATV, AZT and ETR a concordance analysis in susceptibility calls was done using vircoTYPE 4.2 clinical cutoffs [34,35]. AZT and ETR were the RTIs with difference in average squared error between 3F and reference larger than 1.0%. ATV was the drug for which most random divisions were needed to generate the 3F model (Table 4), and one of the PIs with averaged squared error > 7% higher in 3F than in the reference. Click here for file

Additional file 2 **Complexity and performance of 3F and Reference models on genotype-phenotype data sequenced at Virco up to September 2006**. Complexity of the 3F models for the NRTIs, NNRTIs and PIs, and performance on training and test set. The 296 RT mutations found as single term in the RTI 3F models are listed as i) single terms exclusively found in NRTI 3F models, ii) single terms exclusively found in NNRTI 3F models and iii) single terms found in both NRTI and NNRTI 3F models. Click here for file

Additional file 3 **Linear Discriminant Analysis (LDA) for 103N and 181C**. 3F LDA F1 impact on resistance of 103N is largest for NVP: 0.75, then for EFV: 0.63 and then for ETR: 0.09. 3F LDA F1 impact on resistance of 181C is largest for ETR: 0.56, then for EFV: 0.19 and then for NVP: 0.11. LDA cutoff (blue line) is shown to discriminate between samples with wild type at position 103/181 and samples with mutation 103N/181C for which the density histograms are shown. Frequency of wild type (not within a mixture) in LDA data set was 62,010 and 72,643 for positions 103 and 181, respectively. Frequency of mutation (not within a mixture) in LDA data set was 12,012 and 5043 for 103N and 181C, respectively. Click here for file

Additional file 4 **Site Directed Mutants of novel mutations tested for NVP, EFV and ETR**. Fold Change (FC) was calculated as the IC_50 _of the site-directed mutant divided by the IC_50 _of a wild-type laboratory reference strain. All SDMs were measured three times (unless indicated otherwise) and FCs for each of the three measurements are shown. SDMs used as genetic background for evaluating the contribution to resistance of the novel mutations, are given at the top of the file. Noteworthy, the in vitro drug resistance interaction mechanism of the novel mutation and the known NNRTI resistance associated mutations was not always additive: 181F contributed to resensitization to EFV of the 103N mutated virus, 179Y contributed to resensitization to NVP and EFV of the 190A mutated virus. Click here for file

Additional file 5 **K Fold cross-validated stepwise regression using same or different random division before each removal step: ETR model**. Different choices of fold K were evaluated for the ETR model. The goal was to find a linear regression model with better SBC than the reference and at the same time using less parameters. (*A*) When keeping the same random division during the stepwise regression, selection bias resulted in more overfitting, when lowering K. By altering the random division before each removal step, for K = 3 the reference goal SBC was reached with the lowest number of parameters. (*B*) The difference between SBC_CV _and SBC (calculated as *n *ln(*CVPRESS/SSE*)) was found to be larger when lowering the number of folds K, in case a different random division was used before each removal step. (*C*) When lowering K, using a different random division before each removal step resulted in more parameter removals. Whereas for K = 3, a model with the required SBC was found using 700 backward-forward cycles, for K = 2, the model size did not increase fast enough during the stepwise procedure as 2000 backward-forward cycles were not sufficient to reach the goal SBC. Click here for file
