# Supplementary material for: Cross-validated stepwise regression for identification of novel non-nucleoside reverse transcriptase inhibitor resistance associated mutations
Source: BMC Bioinformatics. 2011 Oct 3;12:386. doi: 10.1186/1471-2105-12-386 (PMC3223907; doi:10.1186/1471-2105-12-386)
Supplement: Additional file 1 — Comparison of susceptibility call between 3F and Reference on unseen data for ATV, AZT and ETR. For ATV, AZT and ETR a concordance analysis in susceptibility calls was done using vircoTYPE 4.2 clinical cutoffs [34,35]. AZT and ETR were the RTIs with difference in average squared error between 3F and reference larger than 1.0%. ATV was the drug for which most random divisions were needed to generate the 3F model (Table 4), and one of the PIs with averaged squared error > 7% higher in 3F than in the reference. [file 1471-2105-12-386-S1.PDF]

Comparison of susceptibility call between 3F and Reference on unseen data for ATV, AZT and ETR.

	ATV					AZT					ETR			
		reference					reference					reference		
		MA	RE	MI			MA	RE	MI			MA	RE	MI
	MA	5100	44	0		MA	4685	73	0		MA	4967	312	0
3F	RE	57	1291	129	3F	RE	63	2401	178	3F	RE	294	2366	103
	MI	0	163	1927		MI	0	150	1148		MI	1	92	358
% Concordant			95.49%		% Concordant			94.67%		% Concordant			90.56%	
% Minor discordant			4.51%		% Minor discordant			5.33%		% Minor discordant			9.43%	
% Major discordant			0.00%		% Major discordant			0.00%		% Major discordant			0.01%	

Susceptibility calls using vircoTYPE 4.2 clinical cutoffs: FC ≤ CCO1: MA= maximal response, CCO1 < FC ≤ CCO2: RE= reduced response, FC > CCO2: MI= minimal response.
ATV: CCO1= 2.5, CCO2= 32.5; AZT: CCO1= 1.5, CCO2= 11.4; ETR: CCO1= 1.6, CCO2= 27.6
Minor discordances: MA(3F)-RE(reference), RE-MA, RE-MI or MI-RE.
Major discordances: MA(3F)-MI(reference) or MI-MA.

Genotype with major discordance for ETR between 3F and Reference.

RT Mutations

37L, 41L, 43K/Q, 44D, 49R, 53D, 58N, 64R, 67N, 69A, 74I, 75S, 98S, **101Q**, **108I**, 118I, 123N, 135M, 162D, 169D, 177E, 178L, **179F**, **181C**, 184V, **190A**, 196E, 203K, 210W, 211K, 214F, 215Y, 219D, 223Q, 272A, 277K, 293V, 297A, 322T, 334L, 342F, 356K, 366R, 376S, 377L, 386I, 390R, 395R, 399D

Pheno Fold Change (FC)	72.0

3F

intercept	no mutations	-0.06
single_1	49R	-0.06
single_2	67N	-0.06
single_3	74I	0.08
int_1	**108I & 181C**	0.12
int_2	118I & 214F	-0.14
single_4	178L	0.08
int_3	**179F & 181C**	1.17
single_5	**181C**	0.71
int_4	**181C** & 184V	-0.18
single_6	184V	-0.03
single_7	203K	-0.08
single_8	214F	0.14
single_9	215Y	-0.29
single_10	219D	0.39
single_11	356K	-0.03
single_12	376S	0.05
single_13	386I	-0.10
single_14	395R	-0.07
	log FC	1.62
	FC	41.6

Reference

intercept	no mutations	-0.08
	49R & **190A**	0.11
	49R & 214F	-0.08
single_2	67N	-0.08
	74I & 184V	0.04
	**101Q**	0.06
	**108I**	0.06
int_2	118I & 214F	-0.09
single_4	178L	0.07
	**179F**	0.99
	**179F** & 184V	0.41
	**179F** & 376S	-0.79
	**179F** & 395R	-0.61
single_5	**181C**	0.80
int_4	**181C** & 184V	-0.20
	**181C & 190A**	-0.12
	**181C** & 214F	-0.10
single_6	184V	-0.04
	184V & 223Q	-0.14
single_7	203K	-0.06
single_8	214F	0.16
single_9	215Y	-0.31
single_10	219D	0.32
single_11	356K	-0.04
single_12	376S	0.06
single_13	386I	-0.11
single_14	395R	-0.08
	399D	0.03
	log FC	0.19
	FC	1.55

Known NNRTI mutations are indicated in bold. Pheno call is resistant (FC > BCO of 3.2) and 3F calls Minimal Response (FC > CCO2 of 27.6) for this sample. The Reference call is discordant by predicting Maximal Response (FC ≤ 1.6). In the Reference linear model the overall contribution for mutation 179F is zero, taking the interactions into account (0.99 + 0.41 – 0.79 – 0.61). Linear model coefficients (log FC) are summed to calculate the genotype resistance (log FC).
