# Supplementary material for: Cross-validated stepwise regression for identification of novel non-nucleoside reverse transcriptase inhibitor resistance associated mutations
Source: BMC Bioinformatics. 2011 Oct 3;12:386. doi: 10.1186/1471-2105-12-386 (PMC3223907; doi:10.1186/1471-2105-12-386)
Supplement: Additional file 2 — Complexity and performance of 3F and Reference models on genotype-phenotype data sequenced at Virco up to September 2006. Complexity of the 3F models for the NRTIs, NNRTIs and PIs, and performance on training and test set. The 296 RT mutations found as single term in the RTI 3F models are listed as i) single terms exclusively found in NRTI 3F models, ii) single terms exclusively found in NNRTI 3F models and iii) single terms found in both NRTI and NNRTI 3F models. [file 1471-2105-12-386-S2.PDF]

Complexity and performance of 3F and Reference models on genotype-phenotype data sequenced at Virco up to September 2006.

	Reference Sep 2006						3F Sep 2006[a]					Unseen data Sep 2006 - Dec 2008		
drug	*N train*	single[b] terms	int[c] terms	mut[d]	AIC	SBC	single terms	int terms	mut	AIC	SBC	*N test*	ase[e] Refe-rence	ase 3F
							Nucleoside RT inhibitors[f]							
AZT	45734	80	108	123	-86456	-84806	66	77	102	-86138	-84880	8698	0.091	0.093
3TC	47422	59	64	70	-118357	-117270	43	52	45	-118170	-117329	8733	0.059	0.059
ddI	47269	49	21	62	-111503	-110881	50	25	54	-111500	-110834	8746	0.054	0.054
d4T	47235	47	34	68	-114146	-113427	54	20	60	-114073	-113416	8749	0.050	0.050
ABC	45908	71	46	90	-113501	-112470	63	24	68	-113260	-112492	8749	0.048	0.048
FTC	16440	31	35	46	-37848	-37331	34	34	36	-37894	-37362	8722	0.086	0.086
TDF	31640	64	91	110	-92172	-90867	79	83	111	-92097	-90734	8757	0.065	0.064
							Nonnucleoside RT inhibitors[g]							
NVP	47400	124	190	142	-97887	-95126	103	148	110	-97418	-95209	8729	0.101	0.100
EFV	46054	191	167	211	-84185	-81048	126	101	142	-82822	-80830	8687	0.266	0.264
ETR	18166	122	158	160	-39840	-37646	94	72	119	-38684	-37380	8493	0.126	0.124
							Protease inhibitors[h]							
IDV	47151	91	172	122	-115207	-112894	100	167	124	-115460	-113112	8710	0.066	0.067
NFV	47473	105	206	132	-122581	-119846	103	192	112	-122499	-119904	8710	0.064	0.066
SQV	47446	86	187	120	-114182	-111780	88	183	113	-114078	-111693	8614	0.068	0.073
APV	43997	79	135	107	-115584	-113715	84	136	100	-115559	-113638	8709	0.058	0.059
LPV	25797	66	181	98	-74916	-72893	76	132	93	-74442	-72737	8720	0.111	0.116
ATV	16847	61	137	95	-41290	-39752	73	142	102	-41052	-39382	8711	0.084	0.090
TPV	11869	53	97	90	-36270	-35155	63	63	87	-35711	-34774	8734	0.056	0.056
DRV	16471	69	65	90	-36081	-35040	58	66	86	-36362	-35399	8720	0.091	0.091
NRTI		194	310	226			172	247	199					
NNRTI		282	454	300			196	280	222					
RTI[i]		377	750	407			296[j]	524[k]	336					
PI		178	831	198			170	781	189					

[a]July-September genotype-phenotype 2006 data was used as validation set for 3F.
[b]Number of single terms (first order effects) in model.
[c]Number of interaction terms in model.
[d]Number of mutations in model.
[e]Average squared error on unseen genotype-phenotype data collected between September 2006 and December 2008.
[f]NRTI= AZT, 3TC, ddI, d4T, ABC, FTC and TDF.
[g]NNRTI= NVP, EFV and ETR.
[h]PI= IDV, NFV, SQV, APV, LPV, ATV, TPV, DRV.
[i]RTI = NRTI and NNRTI.
[j]Single terms in one or more RTI 3F models: 296 (81 single terms less than in the reference models)

i. Single terms in one or more NRTI 3F models (100): 3G, 4S, 6D, 7P, 13T, 28K, 33G, 35A/L/M/T, 36A/D, 43E/N/Q, 44A/D, 58N, 63V, 64H/N/Y, 65R, 67E/S/T/del, 68R/Y, 69D/E/N/del/ins, 70E/G/N/Q/R/T, 73M, 74V, 75A/I/M/S/T, 77L, 97S, 116Y, 132V, 151K, 157A, 164L, 172K, 173K, 174K, 200K, 201I, 203Q/T, 207A/E, 210W, 211K/S, 215C/E/I/N/V, 219T/W, 228H, 240P, 242H/L, 245K, 249Q, 284K, 291D, 296S, 313T, 328D, 334H/N, 335D/E, 339W, 355G, 357K, 359S, 360T/V, 364E.
ii. Single terms in one or more NNRTI 3F models (124): 2V, 49R, 69G, 100V, 101A/D/N, 102E/L, 103H/T, 106L, 108I, 109I, 118I, 134N, 135K/M/R/T/V, 138A/G/K/Q, 139K/R, 142T, 158S, 162A/C, 165I/L, 166R, 169D, 171Y, 173T, 178M, 179A/D/E/F/I/M/N/Y, 181F/G/S/V, 188C/F/H, 189I, 190C/E/Q/S/T, 195T, 202T/V, 204K, 206I, 208F, 219D/H, 221C/L, 224D, 225H, 227L, 230I, 234I, 237E, 238N/T, 240K, 241M, 257L, 272A, 283I, 286A, 312D, 313K, 317A, 318F, 324E, 325I, 333E, 335S, 348T, 356K, 357T, 358K, 365I, 366R, 369I, 370A/D/G, 375V, 376A/G/I/S/V, 377L/R/S, 379C/G, 381I, 382L/T, 385R, 386A/V, 393M, 399D/G, 400I/L/S.
iii. Single terms in common between any NRTI and NNRTI 3F models (72): 20R, 21I, 31L, 35I, 40F, 50T, 60I, 62V, 67G/H/N, 68G, 69S, 74I, 75L, 83K, 88C, 90I, 98G/S, 100I, 101E/H/P/Q, 103N/S, 106A/I/M, 115F, 135L, 142V, 151M, 162H/Y, 177E, 178L, 181C/I, 184I/V, 188L, 190A, 196E, 203K, 208Y, 214F, 215D/F/S/Y, 218E, 219E/N/Q/R, 221Y, 223E, 228R, 230L, 244V, 245T, 297K, 329L, 348I, 358G, 369V, 371V, 386I, 395R, 400A.

[k]Three interaction terms in common between AZT and NNRTI: 103N&181C for AZT, EFV and NVP; 103N&184V for AZT and EFV; 215Y&219E for AZT and ETR.
