# Supplementary figures and images for: Cross-validated stepwise regression for identification of novel non-nucleoside reverse transcriptase inhibitor resistance associated mutations
Source: BMC Bioinformatics. 2011 Oct 3;12:386. doi: 10.1186/1471-2105-12-386 (PMC3223907; doi:10.1186/1471-2105-12-386)

NVP

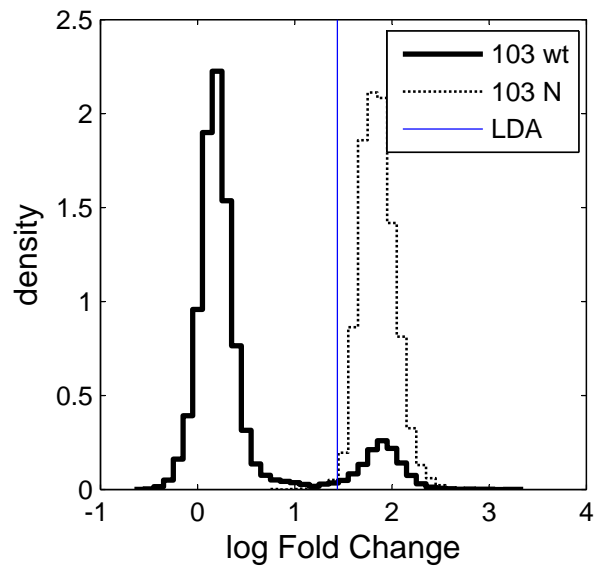

EFV

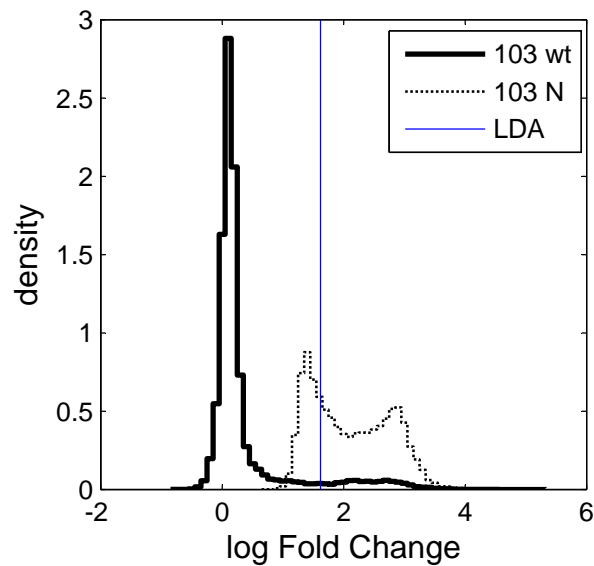

ETR

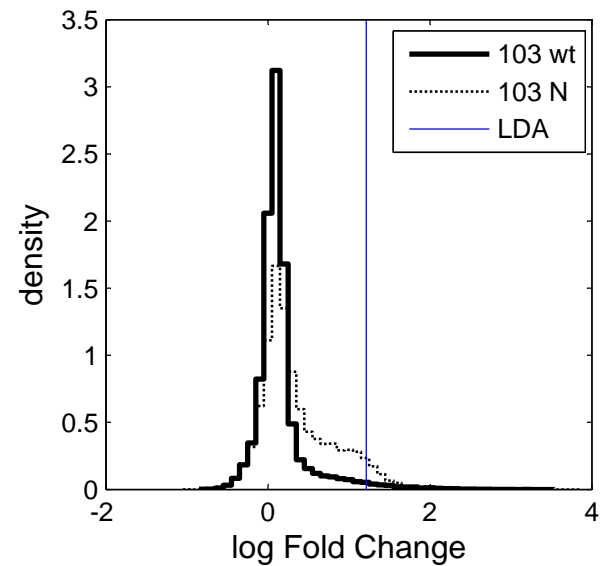

NVP

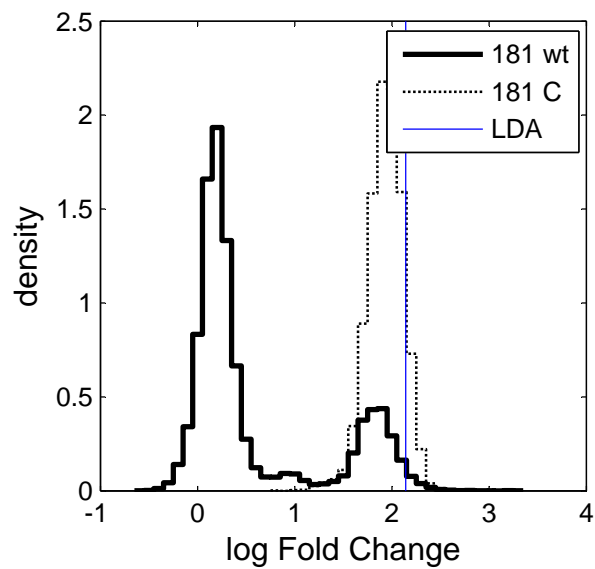

EFV

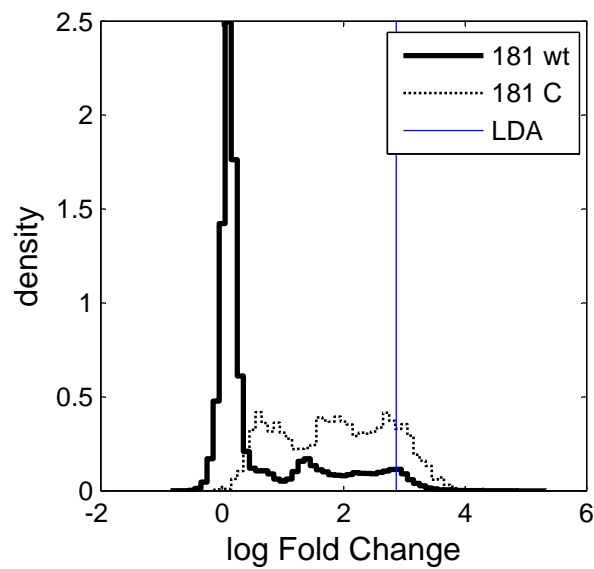

ETR

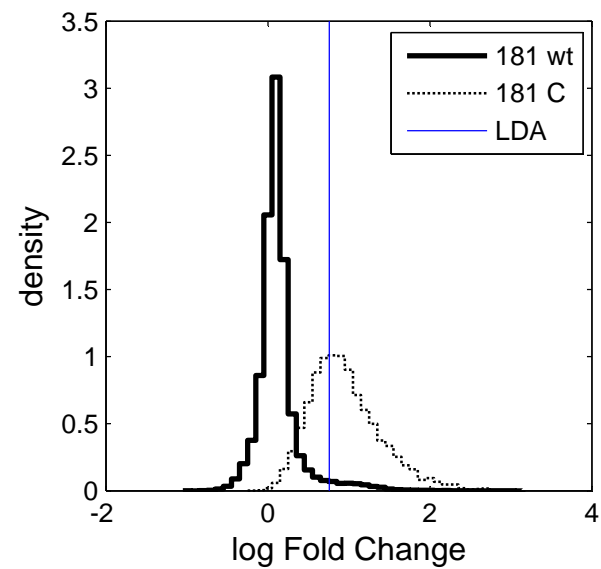

Supplement: Additional file 3 — Linear Discriminant Analysis (LDA) for 103N and 181C. 3F LDA F1 impact on resistance of 103N is largest for NVP: 0.75, then for EFV: 0.63 and then for ETR: 0.09. 3F LDA F1 impact on resistance of 181C is largest for ETR: 0.56, then for EFV: 0.19 and then for NVP: 0.11. LDA cutoff (blue line) is shown to discriminate between samples with wild type at position 103/181 and samples with mutation 103N/181C for which the density histograms are shown. Frequency of wild type (not within a mixture) in LDA data set was 62,010 and 72,643 for positions 103 and 181, respectively. Frequency of mutation (not within a mixture) in LDA data set was 12,012 and 5043 for 103N and 181C, respectively. [file 1471-2105-12-386-S3.PDF]
