# Supplementary material for: Cross-validated stepwise regression for identification of novel non-nucleoside reverse transcriptase inhibitor resistance associated mutations
Source: BMC Bioinformatics. 2011 Oct 3;12:386. doi: 10.1186/1471-2105-12-386 (PMC3223907; doi:10.1186/1471-2105-12-386)
Supplement: Additional file 4 — Site Directed Mutants of novel mutations tested for NVP, EFV and ETR. Fold Change (FC) was calculated as the IC50 of the site-directed mutant divided by the IC50 of a wild-type laboratory reference strain. All SDMs were measured three times (unless indicated otherwise) and FCs for each of the three measurements are shown. SDMs used as genetic background for evaluating the contribution to resistance of the novel mutations, are given at the top of the file. Noteworthy, the in vitro drug resistance interaction mechanism of the novel mutation and the known NNRTI resistance associated mutations was not always additive: 181F contributed to resensitization to EFV of the 103N mutated virus, 179Y contributed to resensitization to NVP and EFV of the 190A mutated virus. [file 1471-2105-12-386-S4.PDF]

Site Directed Mutants of novel mutations tested for NVP, EFV and ETR.

Known NNRTI resistance associated mutations	NVP n = 3 measurements (Fold Change in IC_{50})			EFV n = 3 measurements (Fold Change in IC_{50})			ETR n = 3 measurements (Fold Change in IC_{50})		
100I	7.3	3.8	9.5	17.0	29.6	29.5	1.0	1.6	1.5
103N	> 51.7	> 54.2	35.4	19.5	15.1	12.5	1.1	0.9	0.6
138K[a]	1.7			2.2			2.4		
181C[b]	109.0	60.0		1.4	1.4		3.1	3.5	
181I[b]	> 77.3	> 65.7		1.3	1.8		8.8	13.8	
188L[c]	> 22.7	> 21.7	> 22.7	84.9	88.7		1.3	1.3	0.7
190A	>86.6	>89.2	145.5	4.1	8.1	4.8	1.0	0.9	0.5
90I+181I	> 494.3	> 501.3	> 402.0	3.7	4.1	1.1	36.8	45.4	18.3
100I+138K	1.9	1.1	1.5	5.2	3.0	4.0	2.2	2.4	3.2
103N+181C	> 85.9	> 85.9	> 85.9	28.4	23.1	38.7	4.2	1.7	3.2
108I+181C	> 22.7	> 22.7	> 22.7	3.6	3.5	5.8	1.8	3.1	3.2
181C+188L	> 22.7	> 22.7	> 22.7	205.0	132.4	111.2	5.1	5.2	6.0

Novel NNRTI resistance associated mutations tested as single mutation or in combination	NVP n = 3 measurements (Fold Change in IC_{50})			EFV n = 3 measurements (Fold Change in IC_{50})			ETR n = 3 measurements (Fold Change in IC_{50})		
100V[d]	6.4			18.3	9.0		1.1	1.2	
101A	13.4	8.8	12.8	4.4	5.6	4.1	1.8	1.8	1.5
101D	13.3	17.0	18.9	6.8	6.3	5.7	1.0	1.2	1.3
101N[a]	5.0			2.8			0.8		
102L	2.3	2.9	2.9	1.1	1.2	0.5	0.3	0.4	0.3
102L+188L	> 22.7	> 22.7	> 22.7	410.7	230.5	220.0	2.6	2.2	3.3
102L+108I+181C	> 23.5	> 23.5	> 23.5	1.8	3.7	3.9	4.5	6.2	5.3
106L	0.6	0.6	0.5	0.9	0.7	1.1	0.3	0.2	0.3
134N	1.5	2.1	1.2	0.7	0.6	0.7	0.9	0.8	0.9
138A	2.2	4.9	2.4	1.4	3.6	1.2	2.8	3.5	3.0
138A+181I	> 449.2	> 402.0	> 413.7	2.0	2.7	3.8	43.4	56.4	70.1
138A+90I+181I	> 501.3	> 448.7	> 413.7	2.3	2.1	1.5	141.8	87.6	80.3
139K	4.8	5.8	4.4	1.2	2.3	1.9	2.4	3.0	2.8
139K+100I	12.5	8.6	9.5	18.3	38.7	37.1	1.2	1.8	3.9
139K+138K[e]				3.0	3.7	2.3	4.6	4.8	2.8
139K+100I+138K[e]				9.9	5.4	6.7	1.5	3.3	3.3
139R	7.3	7.3	6.4	1.5	2.2	2.7	1.2	1.2	1.0
139R+103N+181C	> 22.7	> 21.8	> 22.7	60.2	77.0	61.2	7.0	7.4	5.6
139R+181C+188L[c]	> 22.7	> 22.7	> 22.7	444.0	397.5		20.9	33.9	26.6
179M	1.2	2.3	0.8	0.3	0.3	0.4	0.4	0.9	0.5
179N	1.9	1.6	1.2	0.9	1.1	1.2	0.8	0.8	0.6
179Y	0.2	0.2	0.5	< 0.2	< 0.2	0.2	0.2	< 0.1	0.2
179Y+190A	12.4	22.0	16.5	1.0	1.3	0.8	< 0.1	< 0.1	< 0.1
181F	1.5	2.0	1.1	0.4	0.8	0.4	0.4	0.7	0.4
181F+103N	> 35.6	23.5	23.7	4.3	5.8	8.2	0.3	0.2	0.2
181G	30.1	38.3	> 37.2	1.6	1.3	1.1	1.5	0.9	1.2
181G+103N	> 54.2	> 49.3	> 54.2	53.2	67.3	78.5	3.0	2.4	2.7
181G+190A	> 54.2	> 54.2	> 54.2	18.5	24.9	24.2	1.3	1.0	1.7
188F	1.4	1.7	1.9	0.5	0.3	0.4	0.6	0.4	0.2
188F+103N[c]	> 61.8	> 51.9	> 61.8	28.3	17.4		0.7	0.8	0.7
190T	> 67.4	> 67.4	> 67.4	11.3	7.8	14.9	0.6	0.7	0.7
206I	1.0	1.5	1.7	0.4	0.6	0.7	0.5	0.5	0.9
219D	3.7	3.8	1.4	1.3	2.2	1.3	1.1	2.0	0.4
219D+103N+181C	> 79.6	> 79.6	> 76.7	116.6	72.9	48.6	15.5	20.2	21.4
219H	2.1	2.2	1.3	1.1	1.0	1.8	1.8	1.7	1.1
219H+103N+181C	> 79.6	> 79.6	> 79.6	92.6	23.7	49.0	14.5	16.1	11.6
221L	5.0	4.3	4.2	1.6	1.5	1.6	1.1	1.1	1.1
221L+103N[c]	>79.6	>76.7	>76.7	47.3	58.8		0.8	0.9	1.1
234I	0.6	1.0	0.6	2.3	1.9	1.6	0.9	0.9	1.1
234I+181C	> 55.8	33.7	27.6	3.6	2.9	3.6	9.4	4.3	3.6
241M	4.7	5.4	5.6	1.4	1.0	1.8	0.8	1.0	1.2
357T[f]	0.9			1.2					
376S	2.8	3.4	2.9	2.0	1.5	1.8	2.1	1.8	1.6
382T	2.5	2.4	6.7	0.9	1.5	1.7	0.7	2.4	1.4
386A[g]				1.6			0.5		

[a] n = 1 measurement for all drugs.
[b] n = 2 measurements for all drugs.
[c] n = 2 measurements for EFV.
[d] n = 1 measurement for NVP, n = 2 measurements for EFV and ETR.
[e] no measurements for NVP.
[f] n = 1 measurement for NVP and EFV, no measurements for ETR.
[g] no measurements for NVP, n = 1 measurement for EFV and ETR.
