# Supplementary material for: Cross-validated stepwise regression for identification of novel non-nucleoside reverse transcriptase inhibitor resistance associated mutations
Source: BMC Bioinformatics. 2011 Oct 3;12:386. doi: 10.1186/1471-2105-12-386 (PMC3223907; doi:10.1186/1471-2105-12-386)
Supplement: Additional file 5 — K Fold cross-validated stepwise regression using same or different random division before each removal step: ETR model. Different choices of fold K were evaluated for the ETR model. The goal was to find a linear regression model with better SBC than the reference and at the same time using less parameters. (A) When keeping the same random division during the stepwise regression, selection bias resulted in more overfitting, when lowering K. By altering the random division before each removal step, for K = 3 the reference goal SBC was reached with the lowest number of parameters. (B) The difference between SBCCV and SBC (calculated as n ln(CVPRESS/SSE)) was found to be larger when lowering the number of folds K, in case a different random division was used before each removal step. (C) When lowering K, using a different random division before each removal step resulted in more parameter removals. Whereas for K = 3, a model with the required SBC was found using 700 backward-forward cycles, for K = 2, the model size did not increase fast enough during the stepwise procedure as 2000 backward-forward cycles were not sufficient to reach the goal SBC. [file 1471-2105-12-386-S5.PDF]

K fold cross-validated stepwise regression using same or different random division before each removal step: ETR model

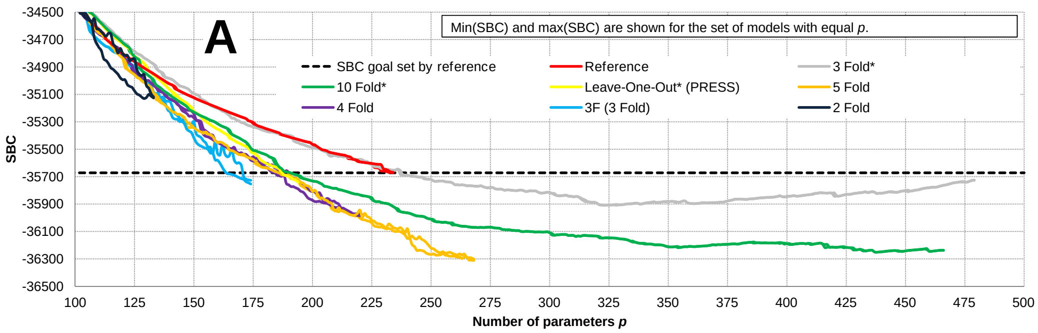

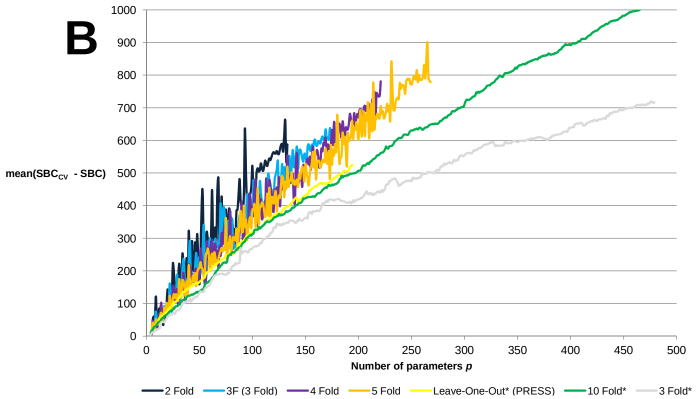

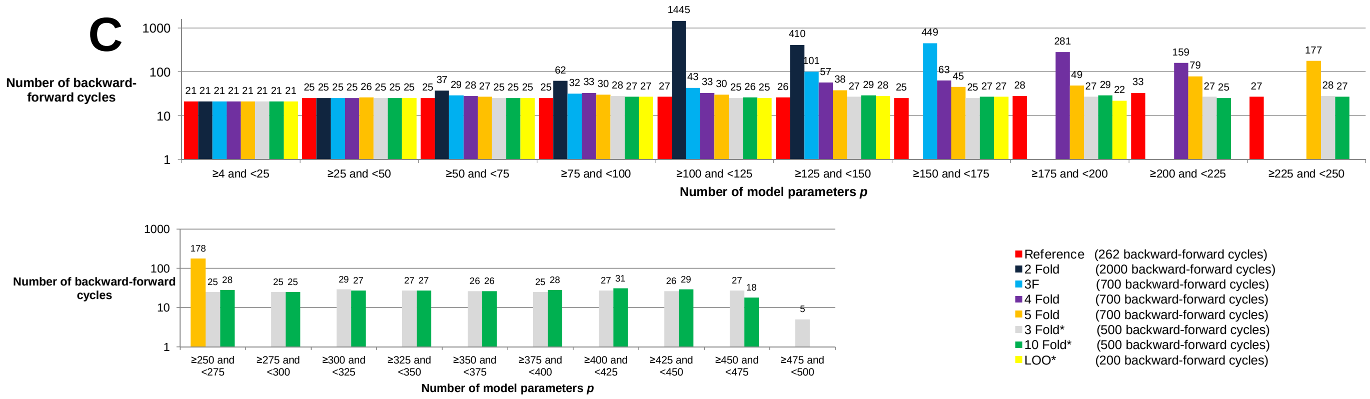

*Using same random division in cross-validation.
